# *Coriandrum sativum* L. Leaf Extract Ameliorates Metabolic Dysfunction-Associated Steatotic Liver Disease by Modulating the AMPK Pathway in High Fat-Fed C57BL/6 Mice

**DOI:** 10.3390/nu16234165

**Published:** 2024-11-30

**Authors:** Min Ji Gu, Yejin Ahn, Yu Ra Lee, Guijae Yoo, Yoonsook Kim, Inwook Choi, Sang Keun Ha, Donghwan Kim

**Affiliations:** 1 Korea Food Research Institute, Wanju-gun 55365, Republic of Korea; gmj9656@kfri.re.kr (M.J.G.); a.yejin@kfri.re.kr (Y.A.); lyr@kfri.re.kr (Y.R.L.); gjyoo@kfri.re.kr (G.Y.); kimyus@kfri.re.kr (Y.K.); choiw@kfri.re.kr (I.C.); skha@kfri.re.kr (S.K.H.); 2 Division of Food Biotechnology, University of Science and Technology, Daejeon 34113, Republic of Korea

**Keywords:** *Coriandrum sativum* L. leaves, metabolic dysfunction-associated fatty liver disease, inflammation, lipid accumulation

## Abstract

Background: Nonalcoholic fatty liver disease (NAFLD) is the most common chronic liver disease. In recent times, the term NAFLD has been modified to metabolic dysfunction-associated steatotic liver disease (MASLD), reflecting its comprehensive scope encompassing a range of metabolic abnormalities. *Coriandrum sativum* L. (CS) is a traditional medicine, although the preventive mechanism of CS extracts remains unclear. Objective: This study evaluated the preventive effects of CS in high-fat diet (HFD)-induced MASLD mice by oral administration of 100 or 200 mg/kg/day of CS extracts for 12 weeks. Results: The major CS extract compounds were chlorogenic acid, caffeic acid, rutin, and isoquercetin. The administration of CS extract suppressed HFD-induced weight gain, liver weight, and the liver/body weight ratio. It improved the mice’s serum biological profiles and suppressed HFD-induced lipid droplet and lipid accumulation by inhibiting lipid accumulation-related gene expression in the liver. It modulated HFD-induced Ampk-Srebp1c pathways and suppressed HFD-induced NF-κB pathway activation in the liver. It regulated inflammation and the AMPK alpha signaling pathway in HFD-fed mice by reducing the accumulation of specific amino acids, leading to the amelioration of fatty liver. Conclusions: The CS extract prevents HFD-induced MASLD and may help prevent or treat MASLD.

## 1. Introduction

Nonalcoholic fatty liver disease (NAFLD) is characterized by excess fat accumulation in the liver, which promotes chronic liver disease and is closely associated with metabolic diseases including obesity, type 2 diabetes, hemochromatosis, and heart disease [1]. A meta-analysis reveals that the incidence and prevalence of NAFLD are continuously increasing and are higher than previous estimates worldwide [2]. Recently, the first nonalcoholic steatohepatitis (NASH) treatment drug was approved by the US Food and Drug Administration [3]. However, there is no approved NAFLD treatment drug, and the main NAFLD treatments include lifestyle changes, weight loss, consumption of a healthy diet, and more exercise [4]. Moreover, the pharmacological agents that demonstrate clinical efficacy in the treatment of liver disease, including NAFLD and NASH, have to undergo further development to enhance their safety profile and target specificity.

A diagnosis of NAFLD is typically made when there is a simple hepatic steatosis, which is defined as the presence of 5% lipid accumulation in the weight of liver tissue, and no other liver disease exists, and there is no evidence of alcoholic causes. Additionally, an NAFLD activity score (NAS) has been proposed, which includes inflammation and hepatocyte ballooning [5]. However, a new diagnostic criterion for metabolic dysfunction-associated fatty liver disease (MAFLD) has recently been discussed. MAFLD is defined by the concomitant presence of fatty liver, type 2 diabetes mellitus, and overweight status or obesity, which differentiates it from NAFLD [6]. After that, regarding the term change from NAFLD, a recent Delphi consensus recommended a revised definition, replacing it with metabolic dysfunction-associated steatotic liver disease (MASLD) [7]. MASLD is a broad term that covers a wide range of metabolic risk factors and can occur in conjunction with other chronic liver diseases. Consequently, to achieve an efficacious treatment of MASLD, which encompasses a multitude of metabolic mechanisms, recent research has focused on the integration of dietary habits, food choice (social nutrition), and lifestyle modifications [8,9]. It is therefore important to consider a wide range of metabolic variables in order to ensure that any intervention is both punctual and efficacious.

*Coriandrum sativum* L. (CS) belongs to the Apiaceae family and is widely used as a cooking ingredient in traditional medicine owing to its beneficial effects [10]. The bioactive compounds in CS include essential oils, fatty acids, tocols, sterols, and carotenoids [11]. The effects of CS on cardiovascular diseases have been discussed and assessed in light of previous studies and reports, where CS was shown to have hypolipidemic, antioxidant, and anti-arrhythmic properties [10]. Computational analysis predicts that CS extracts are potential *Eubacterium rectale* inhibitors that may prevent colorectal cancer through *E. rectale*-induced intestinal inflammation [12]. Polysaccharides from CS extract exhibit antitumor activity and prevent thymus and spleen damage in an H22 hepatoma cell-bearing mouse model [13]. Treatment of prostate cancer PC3 and LNCaP cells with CS extract suppresses cell growth, invasion, and migration, and induces apoptosis [14]. The CS extract suppresses ultraviolet B (UVB)-induced skin photodamage by modulating procollagen type 1 and matrix metalloproteinase-1 expression [15]. Administering 100 mg/kg of ethanol extract from CS seeds to high fat diet (HFD)-induced obese rats shows neuroprotective activity by suppressing oxidative stress and cellular senescence in the brain [16]. The administration of CS extracts with *Foeniculum vulgare* extracts ameliorated HFD-induced hazards such as poor biological parameters in serum and low fertility promotion in female rats [17]. However, the preventive effects of CS on MASLD remain unclear. Therefore, this study aimed to evaluate the preventive effects of CS extract against HFD-induced MASLD in a mouse model.

## 2. Materials and Methods

### 2.1. Sample Preparation

*C. sativum* L. leaves were freeze-dried and crushed into a powder for extraction. The leaves were extracted for 3 h at 50 °C by adding water (1:10 w:v). The procedure was repeated twice, with the remaining residue filtered. Rotary evaporation was carried out for filtrate evaporation and the concentrate was freeze-dried.

### 2.2. High-Performance Liquid Chromatography (HPLC) Analytical Method Conditions

The main compounds in the CS extract were analyzed by HPLC (Waters, Milford, MA, USA) using a YMC Hydrosphere C_18_ column. The mobile phase consisted of acetonitrile with 0.2% formic acid and water with 0.2% formic acid at a flow rate of 1 mL/min. Chlorogenic acid, caffeic acid, rutin, and isoquercetin were detected at 260 nm. The specificity, linearity, limits of detection (LOD), limits of quantification (LOQ), precision, and accuracy of the standard materials were validated based on the International Conference on Harmonization (ICH) guidelines.

### 2.3. Animal Experiments

C57BL/6 male mice (5 weeks) were purchased from Orient Bio Inc. (Kwangju, Korea). Animals were kept under constant conditions (22 ± 2 °C, 51 ± 10% humidity, 12 h light/dark cycle). After one week, the experimental animals were randomly divided into five groups (n = 9): normal chow diet (normal control group; NC), high-fat diet group (60% kcal-fat diet; high-fat control group; HC), high-fat diet with 100 mg/kg/day CS (CS100), high-fat diet with 200 mg/kg/day CS (CS 200, and high-fat diet with 100 mg/kg/day milk thistle extract (positive control; MT)). All mice were given their respective diets by gavage once a day for 12 weeks and treated with the vehicle (distilled water). All the groups had ad libitum access to fresh water. Body weight (BW, g) and food intake (g) were analyzed. After 12 weeks, diets were removed overnight, and the mice were euthanized using isoflurane. Blood was collected and liver tissues were harvested. All animal experiments were granted by the Korea Food Research Institute Animal Care and Use Committee (KFRI-M-20035).

### 2.4. Biochemical Analysis

Serum was collected (3000 rpm for 10 min at 4 °C). Alanine aminotransferase (ALT), aspartate aminotransferase (AST), lactate dehydrogenase (LDH), total cholesterol (TCHO), triglyceride (TG), low-density lipoprotein (LDL) cholesterol, high-density lipoprotein (HDL) cholesterol, and glucose (GLU) were analyzed by automatic blood biochemical test analyzer (AU680, Beckman Coulter, Tokyo, Japan). Insulin levels were analyzed by commercial kit (Fuji Film Co., Tokyo, Japan). The homeostatic index of insulin resistance (HOMA-IR) was measured according to the following HOMA method [18].

### 2.5. Liver Histology

Liver tissues were fixed in 10% formalin to determine histological changes and tissue injury. For stains of hematoxylin and eosin (H&E), fixed tissues were paraffin-embedded, and then slices were sectioned at 4 µm. Additionally, liver tissues were frozen and sectioned for Oil Red O (ORO) staining, and Image J software (Version 1.54g, NIH, Bethesda, MD, USA) was used for calculating the lipid-positive area. All tissue slides were scanned by slide scanner (Motic Easyscan, Motic DS Assistant, Motic, Hong Kong). The NAFLD activity was scored as shown in Table S1. Briefly, the scores ranged from 0 to 3 for steatosis and lobular inflammation, with 0 to 1 indicating mild, 1 to 2 indicating moderate, and 3 indicating severe. The hepatocyte ballooning score was 0–2, and the NAFLD activity score (NAS) was used to quantify each parameter as described in a previous study [19]. Hematoxylin and eosin or ORO-stained slides were scored by an observer blinded to the tissue source.

### 2.6. Quantitative Real-Time PCR

Total RNA was extracted from liver tissue using TRIzol reagent (Ambion, Carlsbad, CA, USA) and cDNA was synthesized using the iScript cDNA Synthesis Kit (Bio-Rad, Hercules, CA, USA). Target gene expression was analyzed using SYBR Green Master Mix (Bio-Rad), and *Gapdh* was used as the endogenous gene. Primers were purchased from Bioneer (Dajeon, Republic of Korea) (Table S2).

### 2.7. Western Blot Analysis

Liver tissues were extracted using a homogenizer (TissueLyser LT, QIAGEN, Hilden, Germany) with RIPA buffer (Cell Signaling Technology, Danvers, MA, USA). Proteins were loaded by 10% SDS-PAGE gels and then transferred onto a nitrocellulose membrane. The membranes were blocked with 5% skim milk for 1 h at room temperature. Subsequently, the membranes were incubated with primary antibodies for more than 4 h. The following antibodies were used: phospho-AMP-activated protein kinase alpha (p-AMPKα), Srebp1c, Scd1, Fas, phospho-nuclear factor kappa-B (p-NFκB), phospho-inhibitor of κB (p-IκB), Interleukin-1β (IL-1β), and tumor necrosis factor-α (TNF-α). The membranes were washed, then incubated with secondary antibodies for 1 h. Protein bands were visualized using a chemiluminescence detection reagent and imaged using an imaging system (ChemiDoc™ XRS + System, Bio-Rad). The densitometry of each band was analyzed using Image Lab software (Version 4.1, Bio-Rad) and normalized to the relative ratio of GAPDH. All the antibodies were purchased from Cell Signaling Technology (Danvers, MA, USA).

### 2.8. Metabolite Analysis in Hepatic Tissue

Sixty mg of liver tissues was homogenized twice with 600 µL of 50% MeOH, and 20 µL of internal standard (10 ppm) was added, then the mixture was centrifuged at 12,000 rpm for 15 min at 4 °C. The supernatant was transferred to a liquid chromatography vial and analyzed using liquid chromatography-tandem mass spectrometry. Chromatography was performed using a Waters ACQUITY™ H Class ultra-performance liquid chromatograph coupled to a Xevo triple quadrupole mass spectrometer system (Waters, Manchester, UK). Compounds were separated by gradient elution (A, 0.1% formic acid in water; B, 0.1% formic acid in acetonitrile) at 0.3 mL/min with ACQUITY ultrahigh performance liquid chromatography (UPLC) using a BEH C18 column (1.7 µm, 2.1 × 100 mm) at 40 °C. The gradient elution system was controlled as follows: 0 min, 5% B; 0–17.5 min, 5–95% B; 17.5–19 min, 95–89% B; 19–19.5 min, 89–5% B. The system was then re-equilibrated at the initial condition (5% B) for 2.5 min before the next sample. The instrument parameters were configured with a capillary voltage of 3 kV, cone voltage of 16 V, extractor voltage of 3 V, source temperature of 150 °C, desolvation temperature of 400 °C, and a desolvation gas flow rate of 800 L/h.

### 2.9. Statistical Analysis

All data are represented as the mean ± standard error of the mean (SEM). Statistical analysis was performed using GraphPad Prism 7.0 (San Diego, CA, USA). One-way analysis of variance (ANOVA) was conducted for multiple comparisons, followed by Dunnett’s post hoc test. Statistical significance was set at *p* < 0.05.

## 3. Results

### 3.1. High-Performance Liquid Chromatography Analysis of Marker Compounds in CS Extracts

**Figure 1:**
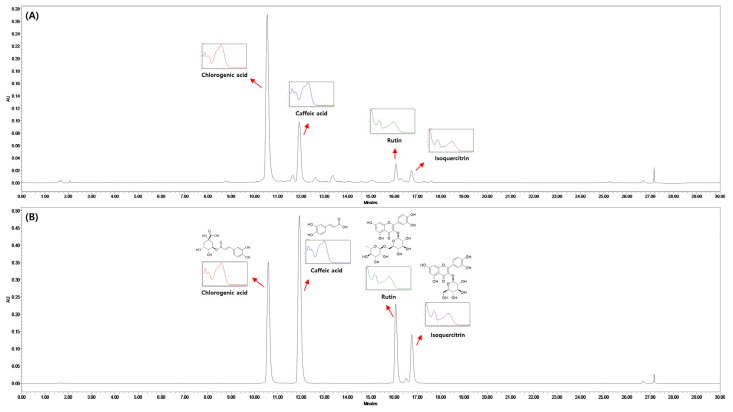
High-performance liquid chromatogram of CS extracts (**A**) and standard solution mixture (**B**). CS, *Coriandrum sativum*.

Chlorogenic acid, caffeic acid, rutin, and isoquercetin were identified as the main compounds in the CS extract, according to HPLC analysis (Figure 1 and Table S3). Chlorogenic acid was the most affluent compound in CS extract (20.65 mg/g). The HPLC analysis method was validated, since all four standard materials showed linearity with R^2^ = 0.9999, and the LOD and LOQ were 6.62–13.01 and 20.06–39.42 μg/mL, respectively (Table S4). Additionally, the relative standard deviation showed precision in the range of 0.85–2.71 and 0.42–1.57% in intra-day and inter-day experiments, respectively, and accuracy was confirmed in the range of 99.13–100.92 and 98.97–101.57%, respectively (Table S5).

### 3.2. The CS Extract Inhibits HFD-Induced Body and Liver Weight in MASLD Mouse Model

**Figure 2:**
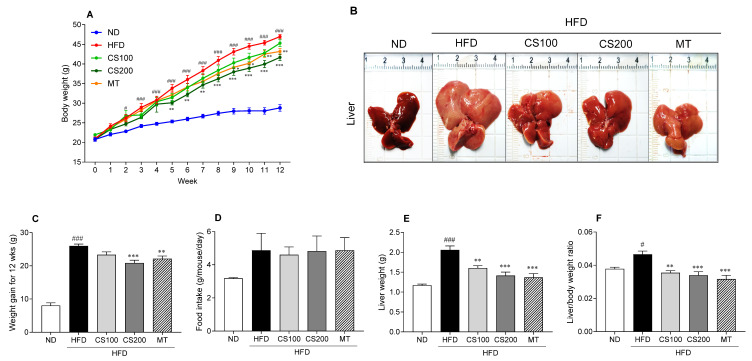
The effect of CS extract treatment on the body and liver weight and morphological change in the liver of HFD-induced MASLD mice. (**A**) Body weight change during the 12 weeks (**B**) Gross appearance of liver tissues (**C**) Weight gain (**D**) Food intake (**E**) Liver weight (**F**) Liver/body weight ratio. Values represent the mean ± standard error of the mean (SEM) (n = 6–9). # *p* < 0.05 and ### *p* < 0.001 vs. ND; * *p* < 0.05, ** *p* < 0.01 and *** *p* < 0.001 vs. HFD. ND: normal diet; HFD: high-fat diet; MASLD: metabolic dysfunction-associated steatotic liver disease; CS: *Coriandrum sativum*; MT: milk thistle.

The body weight in the HFD group was significantly higher than that of the ND group (Figure 2A). In contrast, the administration of 200 mg/kg CS extract markedly suppressed HFD-induced weight gain from the 6th week. Additionally, the MT group showed significant weight loss compared to the HFD from the 9th week. Weight gain significantly increased by 244% over 12 weeks in the HFD group compared with the ND group (Figure 2C). Additionally, there was no significant difference in HFD intake among the groups (Figure 2D). However, the CS200 and MT groups effectively suppressed HFD-induced weight gain. The liver tissue weight significantly increased in the HFD group compared to the ND group (Figure 2B,E); however, the CS extract treatment decreased HFD-induced liver weight compared to the HFD group (Figure 2E). Additionally, significant differences were observed in the liver and body weight ratios between the HFD and control groups (Figure 2F). However, the CS extract-treated group significantly decreased by 25.5 and 27.6%, respectively, compared to the HFD group. These results suggested that the CS extract had an inhibitory effect on HFD-induced body weight gain in body and liver tissues.

### 3.3. The CS Extract Reduces Liver Damage and Metabolic Parameters in the Serum of the HFD-Induced MASLD Mice Model

**Figure 3:**
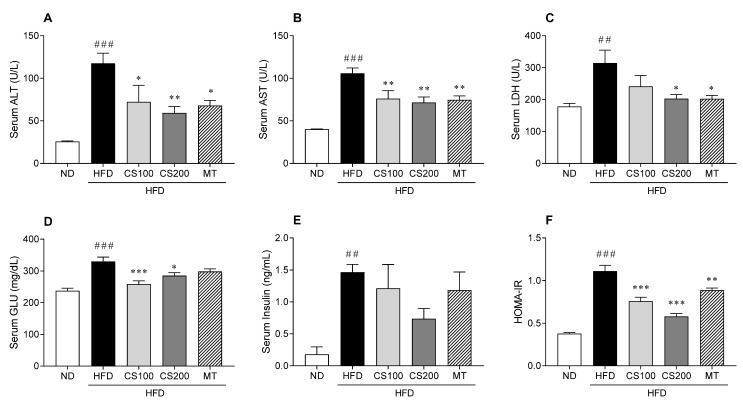
The effect of CS extract treatment on the hepatic function of and metabolic parameters in the serum of HFD-induced MASLD mice. (**A**) Serum ALT (**B**) Serum AST (**C**) Serum LDH (**D**) Serum GLU (**E**) Serum insulin (**F**) HOMA-IR. Values represent the mean ± standard error of the mean (SEM) (n = 6–9). ## *p* < 0.01 and ### *p* < 0.001 vs. ND; * *p* < 0.05, ** *p* < 0.01, and *** *p* < 0.001 vs. HFD. ND: normal diet; HFD: high-fat diet; MASLD: metabolic dysfunction-associated steatotic liver disease; CS: *Coriandrum sativum*; MT: milk thistle; ALT: alanine aminotransferase; AST: aspartate aminotransferase; LDH: lactate dehydrogenase; GLU: glucose; HOMA-IR: homeostatic index of insulin resistance.

The levels of AST, ALT, and LDH (hepatic injury marker parameters) obviously increased in the HFD group by 359, 163, and 76%, respectively, compared to the ND group (Figure 3A–C). However, these parameters decreased following the administration of CS extract in a dose dependent way compared with HFD group. In particular, the 200 mg/kg CS extract treatment group showed significant differences in AST, ALT, and LDH by 53, 32, and 35%, respectively, compared with the HFD group. Additionally, serum glucose and insulin levels were higher in the HFD group than thosein the ND group (Figure 3D,E). However, the serum insulin levels were not significantly different (Figure 3E). Additionally, the HOMA-IR was significantly reduced by 32 and 48% in HFD-induced MASLD mice after administering 100 and 200 mg/kg of CS extract, respectively (Figure 3F).

### 3.4. The CS Extract Alleviates Increases in Serum Lipid Parameters of the HFD-Induced MASLD Mice Model

**Figure 4:**
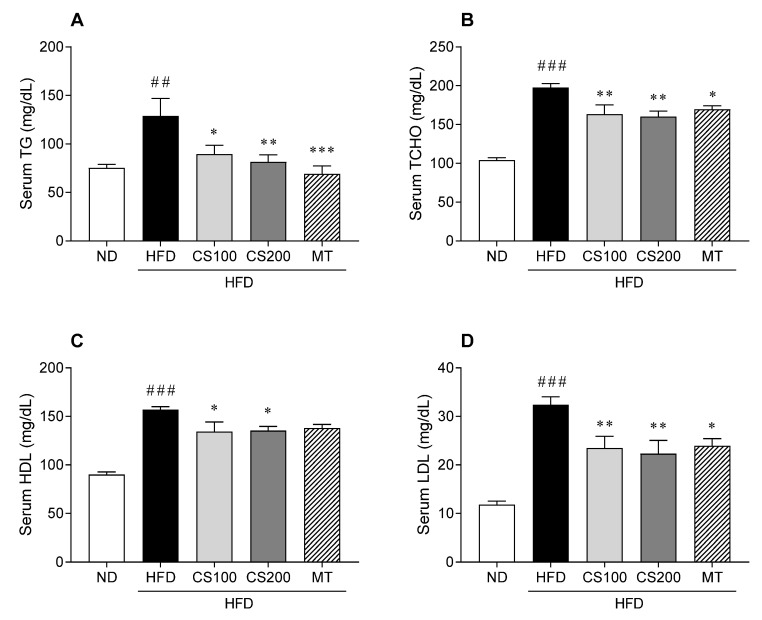
The effect of CS extract treatment on lipid levels in the serum of HFD-induced MASLD mice. (**A**) Serum TG (**B**) Serum TCHO (**C**) Serum HDL (**D**) Serum LDL. Values represent the mean ± standard error of the mean (SEM) (n = 6–9). ## *p* < 0.01 and ### *p* < 0.001 vs. ND; * *p* < 0.05, ** *p* < 0.01, and *** *p* < 0.001 vs. HFD. ND: normal diet; HFD: high-fat diet; MASLD: metabolic dysfunction-associated steatotic liver disease; CS: *Coriandrum sativum*; MT: milk thistle; TG: triglycerides; TCHO: total cholesterol; LDL: low-density lipoprotein cholesterol; HDL: high-density lipoprotein cholesterol.

HFD induced serum TG, TCHO, HDL, and LDL by 90, 79, 74, and 174%, respectively, compared to the ND group (Figure 4). The administration of 100 and 200 mg/kg CS extract significantly reduced the levels of serum TG, TCHO, HDL, and LDL in HFD-induced MASLD mice. However, these parameters decreased following the administration of CS extract compared with the HFD group.

### 3.5. The CS Extract Suppresses Hepatic Damage and Lipid Accumulation in the Liver Tissue of the HFD-Induced MASLD Mice Model

**Figure 5:**
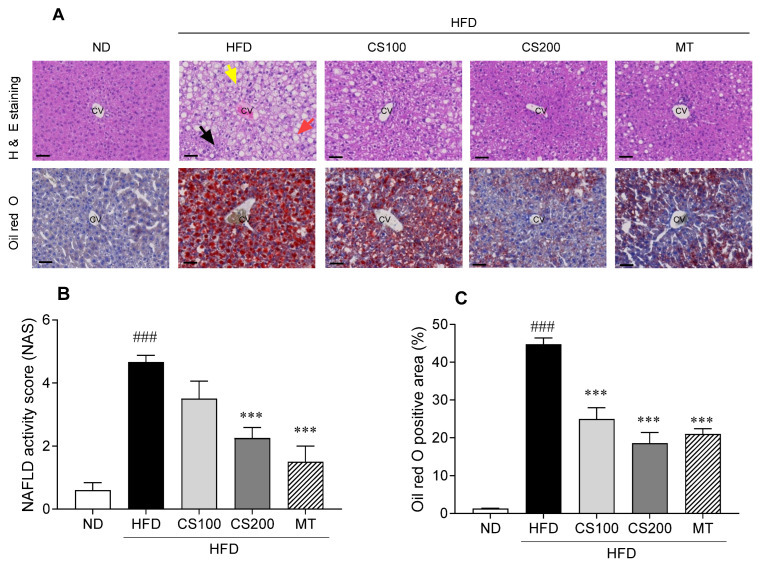
The effect of CS extract treatment on histological alteration in HFD-induced MASLD mice. (**A**) Representative histological results based on hematoxylin and eosin (H&E) and Oil Red O staining (**B**) NAFLD acitivity score (**C**) Oil Red O positive area. Values represent the mean ± standard error of the mean (SEM) (n = 6–9). ### *p* < 0.001 vs. ND; *** *p* < 0.001 vs. HFD. ND: normal diet; HFD: high-fat diet; MASLD: metabolic dysfunction-associated steatotic liver disease; CS: *Coriandrum sativum*; MT: milk thistle; CV: central vein. Scale bar: 50 μm; yellow arrow: balloon cells; red arrow: steatosis; black arrow: inflammation.

Histological changes were observed by H&E and ORO staining to confirm the effect of the CS extract on hepatic damage and lipid accumulation (Figure 5). In H&E analysis, the NAS was analyzed in HFD-induced MASLD mice livers, considering hepatic steatosis, ballooning, and inflammatory infiltrates (Figure S1). The NAS of the HFD group was significantly higher, by 677%, than that of the ND group (Figure 5B); however, the administration of 200 mg/kg CS extract significantly improved the increase in NAS caused by HFD. The fat vacuoles and lipid droplets were widely spread in the HFD group compared with the ND group (Figure 5A). Meanwhile, the administration of 100 and 200 mg/kg CS extract significantly reduced lipid accumulation by 44 and 58%, respectively, compared to the HFD group (Figure 5C).

### 3.6. The CS Extract Regulates Lipid Metabolism-Related mRNA Expression in the Liver Tissue of the HFD-Induced MASLD Mouse Model

**Figure 6:**
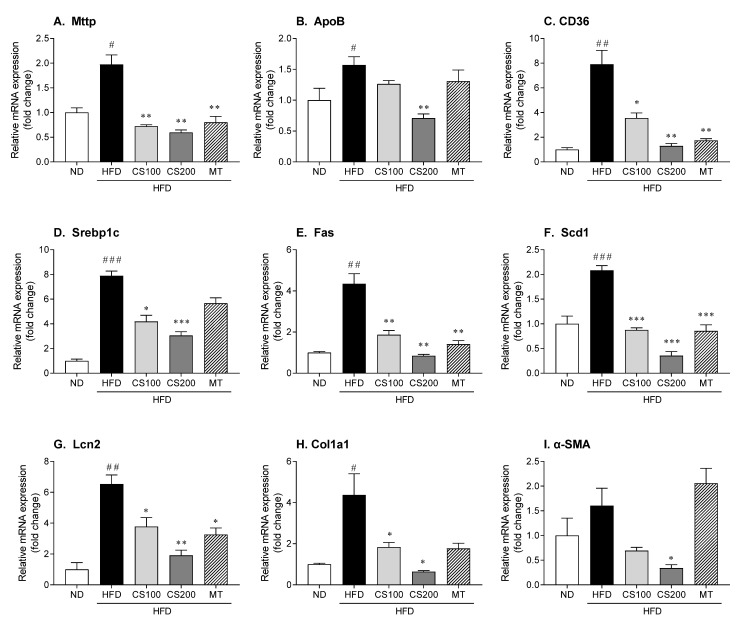
The effect of CS extract treatment on lipid metabolism-related mRNA expression in HFD-induced MASLD mice. (**A**) Mttp: microsomal triglyceride transfer protein (**B**) ApoB: apolipoprotein B (**C**) CD36: cluster of differentiation 36 (**D**) Srebp1c: sterol regulatory element binding protein-1c (**E**) Fas: fatty acid synthesis (**F**) Scd1: stearoyl-CoA desaturase 1 (**G**) Lcn2: lipocalin 2 (**H**) Col1a1: collagen type1 alpha 1 (**I**) α-SMA: alpha smooth muscle actin. Values represent the mean ± standard error of the mean (SEM) (n = 6–9). # *p* < 0.05, ## *p* < 0.01 and ### *p* < 0.001 vs. ND; * *p* < 0.05, ** *p* < 0.01, and *** *p* < 0.001 vs. HFD. ND: normal diet; HFD: high-fat diet; MASLD: metabolic dysfunction-associated steatotic liver disease; CS: *Coriandrum sativum*; MT: milk thistle.

The HFD group significantly increased the expression of *Mttp*, *ApoB*, and *CD36* mRNA hepatic lipid transport-related factors by 97, 57, and 690%, respectively, compared to the ND group, according to qRT-PCR analysis (Figure 6A–C). However, treatment using the CS extract suppressed the expression of *Mttp*, *ApoB*, and *CD36* in a dose-dependent manner, and 200 mg/kg CS extract significantly reduced the expression of these genes by 70, 55, and 84%, respectively. The HFD group also showed significant differences in the gene expression of *Srebp1c*, *Fas*, and *Scd1* lipid synthesis genes in the ND group (Figure 6D–F). However, the administration of 100 and 200 mg/kg CS extract significantly reversed the HFD-induced upregulation of lipid synthesis-related genes. Additionally, the HFD significantly increased *Lcn2* expression by 553%, while the administration of 100 and 200 mg/kg CS extract significantly reduced the increase in *Lcn2* by 42% and 71%, respectively (Figure 6G). *Col1a1* gene expression (a hepatic fibrosis marker) was significantly higher by 337% in the HFD group than that in the ND group (Figure 6H). *α-SMA* expression also tended to increase in the HFD group, but there was no significant difference from the ND group (Figure 6I). Meanwhile, the administration of 200 mg/kg CS extract significantly suppressed the gene expression of *Col1a1* and *α-SMA,* by 85% and 79%, respectively, compared to the HFD group.

### 3.7. The CS Extract Alleviates Lipid Accumulation by Activating the AMPKα Pathway in the Liver Tissue of the HFD-Induced MASLD Mouse Model

**Figure 7:**
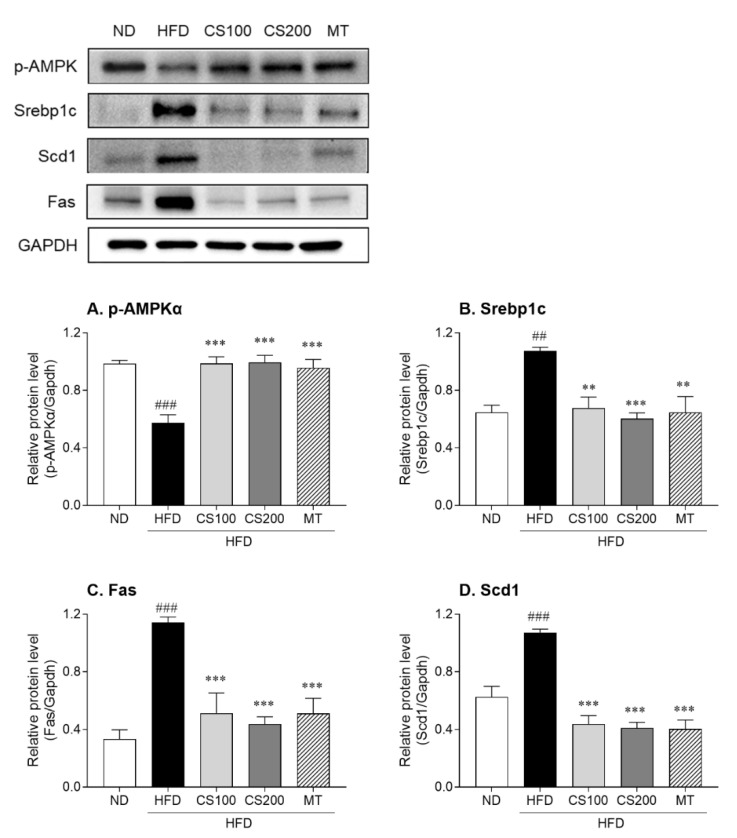
The effect of CS extract treatment on lipid metabolism-related protein levels in HFD-induced MASLD mice. (**A**) p-AMPKα: phospho-AMP-activated protein kinase alpha (**B**) Srebp1c: sterol regulatory element binding protein-1c (**C**) Fas: fatty acid synthase (**D**) Scd1: stearoyl-CoA desaturase-1. Values represent the mean ± standard error of the mean (SEM) (n = 6–9). ## *p* < 0.01 and ### *p* < 0.001 vs. ND; ** *p* < 0.01 and *** *p* < 0.001 vs. HFD. ND: normal diet, HFD: high-fat diet, MASLD: metabolic dysfunction-associated steatotic liver disease, CS: *Coriandrum sativum*, MT: milk thistle.

The HFD significantly inhibited AMPKα phosphorylation by 42% compared to the ND group; however, the administration of 100 and 200 mg/kg CS extract significantly upregulated AMPKα phosphorylation, according to Western blotting analysis (Figure 7A). The protein levels of Srebp1c, Fas, and Scd1 (which are regulated by AMPKα) were significantly elevated by 65, 243, and 71%, respectively (Figure 7B,C). In contrast, the CS extract treatment group showed significantly reversed HFD-induced elevation in the protein levels of Srebp1c, Fas, and Scd1. These results suggested that the CS extract inhibited lipid accumulation in the liver by regulating lipid synthesis factors through AMPK phosphorylation.

### 3.8. The CS Extract Suppresses Inflammation Through Downregulation of the NF-κB Pathway in the Liver Tissue of the HFD-Induced MASLD Mouse Model

**Figure 8:**
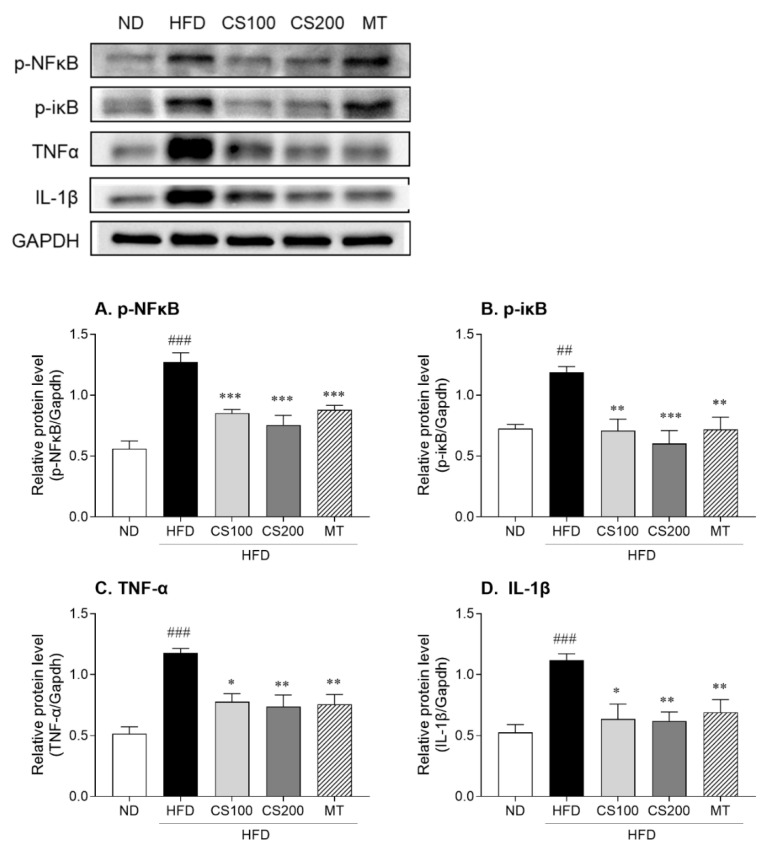
The effect of CS extract treatment on inflammation-related protein levels in HFD-induced MASLD mice. (**A**) p-NFκB: phospho-nuclear factor kappa-B, (**B**) p-IκB: phospho-inhibitor of κB, (**C**) TNF-α: tumor necrosis factor-α, (**D**) IL-1β: Interleukin-1β. Values represent the mean ± standard error of the mean (SEM) (n = 6–9). ## *p* < 0.01 and ### *p* < 0.001 vs. ND; * *p* < 0.05, ** *p* < 0.01, and *** *p* < 0.001 vs. HFD. ND: normal diet; HFD: high-fat diet; MASLD: metabolic dysfunction-associated steatotic liver disease, CS: *Coriandrum sativum*, MT: milk thistle.

NF-κB is a key transcription factor in the inflammatory response that is phosphorylated and translocated into the nucleus, leading to the production of inflammatory cytokines. NF-κB activation is promoted by the phosphorylation of iκB, an inhibitory protein of NF-κB. HFD significantly increased NF-κB and IκB phosphorylation by 127% and 64% compared to the ND group (Figure 8A,B). Meanwhile, the administration of 100 and 200 mg/kg CS extract significantly inhibited NF-κB phosphorylation by 33 and 41%, respectively, compared with the HFD group. Likewise, IκB phosphorylation was significantly lower in the CS extract treatment group than in the HFD group. The protein levels of pro-inflammatory cytokines TNF-α and IL-1β significantly increased by 127 and 112% in the HFD group and the ND group, respectively (Figure 8C,D). The elevation in pro-inflammatory cytokines induced by the HFD was significantly inhibited following the administration of 100 and 200 mg/kg CS extract.

### 3.9. The CS Extract Improves Changes in the Major Metabolites in the Liver Tissue of the HFD-Induced MASLD Mouse Model

**Figure 9:**
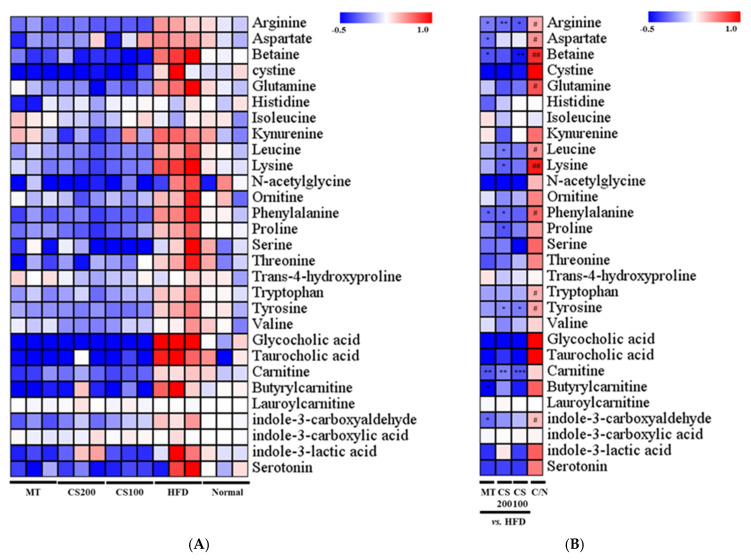
(**A**) Heat map analysis of quantitative metabolite levels in mouse liver tissue. (**B**) Alterations in the fold-changes in each metabolite. The row represents the metabolites, and the column represents the sample. Significantly decreased and significantly increased metabolites are displayed in blue and red, respectively. The brightness of each color is the size of the difference compared to the average value of normal in HFD, and the CS100, CS200, and MT values are the size of the difference compared to the average value of HFD (n = 3). # *p* < 0.05 and ## *p* < 0.01 vs. Normal; * *p* < 0.05, ** *p* < 0.01, and *** *p* < 0.001 vs. HFD.

The liver is a major tissue involved in amino acid catabolism, and significant changes in key amino acid metabolites have been observed in HFD-induced MASLD. Twenty-seven metabolites related to amino acids, bile acids, and carnitine were analyzed to determine the effect of CS extract on metabolic changes in the liver tissue (Figure 9). Among the amino acid metabolites in the HFD group, arginine (0.33-fold), aspartate (0.42-fold), glutamine (0.69-fold), leucine (0.46-fold), lysine (0.90-fold), phenylalanine (0.61-fold), tryptophan (0.31-fold), and tyrosine (0.36-fold) levels were significantly higher than those in the ND group. However, the administration of 100 and 200 mg/kg CS extract significantly decreased the levels of arginine and tyrosine compared to the HFD group. In particular, the CS 200 group showed a significant reduction in branched amino acids (lysine and leucine) by 0.26- and 0.31-fold, respectively, compared to the HFD group. Additionally, bile acids regulate glucose and lipid metabolism, and disruption of the bile acid cycle is closely associated with MASLD. The levels of primary bile acids, taurocholic acid, and glycocholic acid increased in the HFD group compared to the ND group, whereas they decreased in the CS extract-administered group, although the differences were insignificant between the groups. Similarly, betaine synthesized from choline significantly increased by 0.82-fold in the HFD group compared to the ND group; however, treatment with 100 mg/kg CS extract reduced it by 0.46-fold. Carnitine metabolites involved in fatty acid metabolism showed no major differences between the HFD and ND groups; however, carnitine significantly decreased following CS extract treatment compared to the HFD group. Additionally, HFD significantly induced tryptophan-derived indole-3-carboxaldehyde by 0.34-fold compared to ND group. The CS extract treatment group showed a tendency to decrease indole-3-carboxaldehyde and indole-3 lactic acid levels compared to the HFD group, although the difference was insignificant.

## 4. Discussion

NAFLD is a chronic liver disease which promotes various metabolic diseases and affects over one-quarter of the global population [20]. Accumulating evidence shows that NAFLD development is associated with multiple cellular events, including lipid accumulation [20]. Recently, the US Food and Drug Administration (FDA) approved the first NASH treatment drug regimen that effectively suppresses NASH and liver fibrosis in patients [21]. Furthermore, NAFLD terms were replaced in order to elucidate the underlying pathophysiology; an expert panel proposed the designation of ‘metabolic dysfunction-associated fatty liver disease’ (MAFLD) in 2020. Subsequently, in 2023, a broader consensus effort recommended the addition of ‘metabolic dysfunction-associated steatotic liver disease’ (MASLD) as a component of a novel set of terms to cover a broad spectrum of metabolic conditions [22]. However, it is important to reduce the incidence of metabolic disease, including steatotic liver, and the use of natural product-based materials for this purpose has many advantages [11]. *C. sativum* L. is one of the most widely used culinary medicines and is a traditional herbal medicine for a variety of conditions, including inflammation, migraine, hypolipidemia, hypoglycemia, and hypotension, owing to its beneficial effects [23]. In this study, we evaluated the preventive effects of CS extract against HFD-induced MASLD in a mouse model.

CS is known in both cuisine and traditional medicines in Asia, Europe, and India [10]. This study showed that chlorogenic acid, caffeic acid, rutin, and isoquercetin were the major compounds in CS extract, according to HPLC analysis. The capacity of polyphenols to inhibit TG accumulation in hepatocytes has been reported in previous studies [24]. Rutin, a dietary flavonoid commonly found in agricultural products, has been demonstrated to significantly reduce TG synthesis and cholesterol levels in the hepatocytes of fatty liver induced by oleic acid (OA) [25]. In addition, Liu et al. (2018) demonstrated that polyphenolic compounds from blueberries inhibit the TG accumulation in OA-induced lipid accumulation in an in vitro model (HepG2 cells) by caffeic acid or chlorogenic acid [26]. Furthermore, chlorogenic acids suppress cyclophosphamide-induced liver injury in mice [27]. Caffeic acid and rutin ameliorate streptozotocin-induced diabetes in rats [28]. The administration of isoquercetin suppresses NAFLD in mice [29]. These results indicate that CS extract may prevent HFD-induced MASLD in mice.

The HFD-induced NAFLD mouse model reflects human MASLD phenotypes, including insulin resistance, obesity, and high TG levels, that are widely used to evaluate pharmacological drug effects [30]. In this study, the administration of CS extract suppressed HFD-induced weight gain, liver weight, and the liver/body weight ratio in mice. Serum chemistry results showed that the HFD-induced ALT, AST, LDL, and GLU levels were suppressed in mice. The HFD-induced serum insulin levels were insignificantly altered following the administration of CS extract. However, HOMA-IR results showed that the administration of CS extract suppressed HFD-induced insulin resistance. The administration of CS extract suppresses HFD-induced serum TG and LDL levels in mice. High density lipoprotein is considered a beneficial lipoprotein, and HFD promotes serum HDL elevation owing to elevated TCHO levels [31]. This study showed that HFD-induced TCHO and HDL levels were suppressed by CS extract administration. H&E and ORO staining results showed that HFD-induced lipid droplet formation, lipid accumulation, and NAS were ameliorated following the administration of CS extract. These results were similar to those of the MT group as a positive control, suggesting that CS may be involved in the progression of liver damage against lipotoxicity. MT has been used for comparing anti-steatosis activity in in vivo models [32,33], exhibiting the improvement of liver steatosis and inflammation. In addition, metabolic diseases and liver damage were suppressed by reducing steatosis and oxidative stress. The main component of MT is silymarin and other active components, including silybinin and other flavanols, showing that silymarin has potential hepatoprotective effects and may lead to a reduction in fat accumulation and hyperlipidemia in hepatocytes and rats [34,35]. These data indicate that the administration of CS extract improves the biological profiles of serum against HFD-induced stress.

The HFD-induced expression of genes related to lipid export and lipid synthesis (including *Mttp*, *ApoB*, *Cd36*, *Srebp1c*, *Fas*, *Scd1*, and *Lcn2*) was suppressed following the administration of CS extract, according to qRT-PCR analysis. The expression of liver fibrosis-related genes (including *Col1a1* and *α-SMA*) was suppressed following the administration of CS extract. *Mttp* and *ApoB* play a role in the formation of lipid droplets and the secretion of VLDL, which is a process of cellular lipid transfer. This suggests an interaction between the molecules and membranes, whereby the lipid droplets are picked up and then deposited onto other membranes [36]. Previous reports suggested that an elevated hepatic fat content stimulates the VLDL-TG, yet this reaches a plateau above 10% fat, thereby limiting further lipid export [37]. Patients with NAFLD secrete larger, triglyceride-rich VLDL particles without an increase in apoB100 secretion. The inability of large VLDL particles to pass through liver blood vessel pores may result in lipid retention [38]. Despite elevated *ApoB100* and *Mttp* mRNA levels, VLDL secretion remains unaltered, indicating a dysfunction in lipid export [39,40,41]. In this study, the induction of hepatic steatosis was effectively elicited by the high-fat diet, and the mRNA alterations of *Mttp* and *ApoB* were likewise elevated by the reference, indicating that the material may mitigate the symptoms. Furthermore, the AMPK-SREBP1c-SCD1 pathway is important for lipid metabolism in the mouse liver [42]. The HFD suppressed pAMPK protein expression, and HFD-induced Srebp1c, Scd1, and Fas protein expression was suppressed following the administration of CS extract, according to Western blot analysis. These data indicated that CS extract prevented HFD-induced lipid accumulation in the mouse liver. Furthermore, the multiple-hit model for NAFLD and MASLD is considered an acceptable hypothesis, and chronic inflammation accelerates MASLD in fatty livers [43,44]. NF-κB is a transcription factor whose pathway activation promotes an inflammatory response in the liver [45]. This study showed that HFD-induced pNF-κB, p-IκB, TNF-α, and Il1β protein expression were suppressed in mouse livers following the administration of CS extract, according to Western blot analysis. These data suggest that the administration of CS extract suppresses HFD-induced inflammation, which prevents MASLD in the mouse liver.

We quantitatively analyzed 27 metabolites in the liver samples. The liver plays an important role in modulating metabolism in MASLD [46]. In particular, dysregulation of the amino acid metabolism is routinely observed under HFD conditions [47]. Branched-chain amino acid (BCAA) concentrations increase in the plasma of patients with MASLD [48]. Tyrosine metabolism is dysregulated in patients with MASLD [49]. Our results confirmed increased levels of leucine, BCAA, and tyrosine, which are associated with insulin resistance in the MASLD model [50]. Similarly, tryptophan metabolites and enzymes play an important role in MASLD [51]. Our results also showed that tryptophan and indole-3-carboxyaldehyde levels significantly increased in the MASLD group, and that the increased metabolites in the HFD-induced MASLD group returned to normal levels when treated with CS extract. In summary, various metabolic pathways are mediated in the HFD-induced MASLD pathogenesis, and CS extract may help alleviate MASLD. The observed metabolite changes are primarily attributed to MASH improvement, as enhanced MASH status may positively influence metabolic balance and related pathways. However, a limitation of this study is the lack of larger-scale validation. Further large-scale clinical studies involving a greater number of mice or human participants are necessary to comprehensively elucidate the direct and indirect effects of CS extract.

## 5. Conclusions

The CS extract contained chlorogenic acid, caffeic acid, rutin, and isoquercetin. The administration of CS extract suppressed HFD-induced lipid accumulation and inflammation in the livers of mice by lipid accumulation-related gene suppression and fibrosis-related gene suppression. Furthermore, MASLD-related metabolites and amino acids were suppressed by the CS extract. These data suggest that CS extract prevents HFD-induced MASLD and may be used as a preventive or therapeutic agent against MASLD.

## Data Availability

The original contributions presented in the study are included in the article, further inquiries can be directed to the corresponding author.

## Supplementary Materials

The following supporting information can be downloaded at: https://www.mdpi.com/article/10.3390/nu16234165/s1. Table S1. The histological scoring system for NAFLD. Table S2. Primers sequences. Table S3. Contents of four major compounds in CS extract. Table S4. Analytical results of calibration curves, LODs and LOQs of four major compounds. Table S5. Precision and accuracy results of four major compounds. Figure S1. The effect of CS extract treatment on lobular inflammation, hepatocyte expansion, and steatosis scores based on liver histological staining of HFD-induced NAFLD mice. Values are represented as the means ± standard error of the mean (SEM). ### *p* < 0.001 vs. ND; * *p* < 0.05, ** *p* < 0.01, and *** *p* < 0.001 vs. HFD. ND: normal diet; HFD: high-fat diet; NAFLD: non-alcoholic fatty liver disease; CS: *Coriandrum sativum*; MT: milk thistle.
