# Supplementary material for: Coriandrum sativum L. Leaf Extract Ameliorates Metabolic Dysfunction-Associated Steatotic Liver Disease by Modulating the AMPK Pathway in High Fat-Fed C57BL/6 Mice
Source: Nutrients. 2024 Nov 30;16(23):4165. doi: 10.3390/nu16234165 (PMC11644762; doi:10.3390/nu16234165)
Supplement: Supplementary file 1 [file nutrients-16-04165-s001.zip › nutrients-3314603-supplementary.pdf]

Supplementary Data

Table S1. The histological scoring system for NAFLD.

Grade	Steatosis	Lobular inflammation	Ballooning
0	<5%	No foci	None
1	5–33%	<2 foci per 200× field	Few balloon cells
2	>33%–66%	2–4 foci per 200× field	Many cells/prominent ballooning
3	>66%	>4foci per 200× field	

NAFLD, non-alcoholic fatty liver disease.

Table S2. Primers sequences.

	Forward	Reverse
**ApoB**	GGTGTATGGCTTCAACCCTGA	GCTTGAGTTCGTACCTGGACA
**Cd36**	GATTAATGGCACAGACGCAGC	CAGATCCGAACACAGCGTAGA
**Col1a1**	TAGGCCATTGTGTATGCAGC	ACATGTTCAGCTTTGTGGACC
**Fas**	AAGTTGCCCGAGTCAGAGAACC	ATCCATAGAGCCCAGCCTTCCATC
**Lcn2**	TCTGTCCCCACCGACCAAT	GGAAAGATGGAGTGGCAGACA
**Gapdh**	GACTTCAACAGCAACTCCCAC	TCCACCACCCTGTTGCTGTA
**Mttp**	CCCACTCAGGCAATTCGAGA	TGATGGAGGGGGAGTTCACA
**Scd1**	GTTTCCAAGCGCAGTTCCG	TGGAGATCTCTTGGAGCATGTG
**a-SMA**	GACCCTGAAGTATCCGATAGAACA	CACGCGAAGCTCGTTATAGAAG
**Srebp1c**	GGAGCCATGGATTGCACATT	GCTTCCAGAGAGGAGGCCAG

Table S3. Contents of four major compounds in CS extract.

Analyte	Contents (mg/g of dried weight)
Chlorogenic acid	20.65±0.13
Caffeic acid	5.07±0.05
Rutin	3.42±0.01
Isoquercetin	2.99±0.02

Values are represented means ± standard deviation. CS, *Coriandrum sativum*.

Table S4. Analytical results of calibration curves, LODs and LOQs of four major compounds.

Analyte	Regression Equation	Correlation Coefficient (R^2)	Linear range (μg/mL)	LOD[a] (μg/mL)	LOQ[b] (μg/mL)
Chlorogenic acid	y = 2303.49x – 3539.38	0.9999	7.8-250	13.01	39.42
Caffeic acid	y = 3617.48x – 2789.63	0.9999	7.8-250	7.70	23.35
Rutin	y =891.80x – 1737.69	0.9999	7.8-250	6.62	20.06
Isoquercetin	y = 1373.29x – 2443.11	0.9999	7.8-250	8.08	24.51

LOD[a]: limits of detection, LOQ[b]: limit of quantification.

Table S5. Precision and accuracy results of four major compounds.

Analyte	Concentration (μg/mL)	Intra-day (n=3)			Inter-day (n=3)		
		Mean (μg/mL)	RSD (%)	Accuracy (%)	Mean (μg/mL)	RSD (%)	Accuracy (%)
Chlorogenic acid	78.125	77.80	0.93	99.59	77.33	0.42	98.97
	156.25	156.02	0.79	99.86	156.27	0.94	100.02
	312.5	313.30	0.95	100.25	311.73	1.02	99.75
Caffeic acid	78.125	78.85	1.27	100.92	77.71	0.84	99.46
	156.25	156.37	1.08	100.08	155.85	0.79	99.75
	312.5	312.42	1.01	99.97	310.40	1.05	99.32
Rutin	78.125	78.58	2.71	100.58	77.84	1.34	99.63
	156.25	156.37	0.98	100.07	156.83	0.68	100.37
	312.5	313.26	1.08	100.24	311.73	1.08	99.75
Isoquercetin	78.125	77.73	1.50	99.49	78.07	1.57	99.93
	156.25	154.91	1.36	99.13	158.75	0.80	101.57
	312.5	314.03	0.85	100.49	314.10	1.21	100.51

RSD, relative standard deviation.

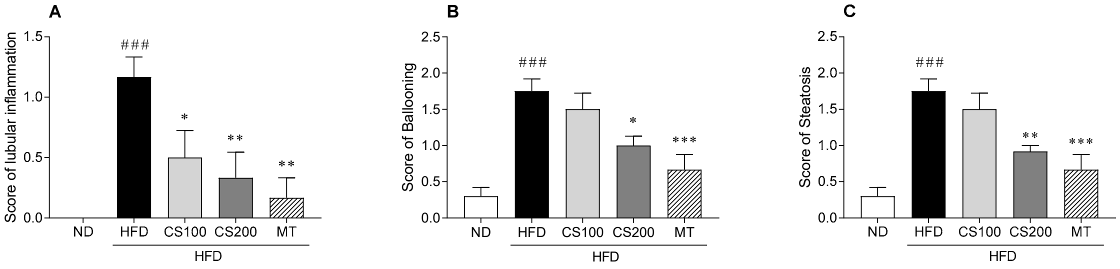

Figure S1. The effect of CS extract treatment on lobular inflammation, hepatocyte expansion, and steatosis scores based on liver histological staining of HFD-induced NAFLD mice. Values are represented as the means ± standard error of the mean (SEM). ### $p < 0.001$ vs. ND; * $p < 0.05$, ** $p < 0.01$, and *** $p < 0.001$ vs. HFD. ND: normal diet; HFD: high-fat diet; NAFLD: non-alcoholic fatty liver disease; CS: *Coriandrum sativum*; MT: milk thistle.
